# Measurement of Left Atrial Pressure is a Good Predictor of Freedom From Atrial Fibrillation

**DOI:** 10.1016/s0972-6292(16)30774-4

**Published:** 2014-07-15

**Authors:** Leonard Bergau, Dirk Vollmann, Lars Luthje, Jan Martin Sohns, Joachim Seegers, Christian Sohns, Markus Zabel

**Affiliations:** 1 Dept. of Cardiology and Pneumology, Heart Center, Georg-August-University of Gottingen, Gottingen, Germany; 2 Dept. of Radiology, Georg-August-University of Gottingen, Gottingen, Germany

**Keywords:** atrial fibrillation, pulmonary vein ablation, predictors, left atrial pressure, remodeling, left atrial volume

## Abstract

**Background:**

It is suggested that an elevated left atrial pressure (LAP) promotes ectopic beats emanating in the pulmonary veins (PVs) and that LAP might be a marker for structural remodeling. This study aimed to identify if the quantification of LAP correlates with structural changes of the LA and may therefore be associated with outcomes following pulmonary vein isolation (PVI).

**Methods:**

We analysed data from 120 patients, referred to PVI due to drug-refractory atrial fibrillation (AF) (age 63±8; 57% men). The maximum (mLAP) and mean LAP (meLAP) were measured after transseptal puncture.

**Results and Conclusions:**

Within a mean follow-up of 303±95 days, 60% of the patients maintained in sinus rhythm after the initial procedure and 78% after repeated PVI. Performing univariate Cox-regression analysis, type of AF, LA-volume (LAV), mLAP and the meLAP were significant predictors of recurrence after PVI (p=0.03; p=0.001; p=0.01). In multivariate analysis mLAP>18mmHg, LAV>100 ml and the presence of persistent AF were significant predictors (p=0.001; p=0.019; p=0.017). The mLAP >18 mmHg was associated with a hazard ratio of 3.8. Analyzing receiver-operator characteristics, the area under the curve for mLAP was 0.75 (p<0.01). mLAP >18 mmHg predicts recurrence with a sensitivity of 77 % and specificity of 60 %. There was a linear correlation between the LAV from MDCT and mLAP (p = 0.01, R2 = 0.61). The mLAP measured invasively displays a significant predictor for AF recurrence after PVI. There is a good correlation between LAP and LAV and both factors may be useful to quantify LA remodeling.

## Introduction

Atrial Fibrillation (AF) is the most common sustained arrhythmia worldwide with a raising prevalence in the elderly patients. [1] AF is regularly associated with decreased quality of life as well as increased morbidity and mortality [2]. In recent years, catheter based ablation for pulmonary vein isolation (PVI) evolved to be the therapy of choice for treatment of drug refractory AF. Although PVI is successful in most of the patients, the long term success rates vary [3,4]. Long-term efficacy of PVI is depended of multiple factors and still difficult to predict for an individual patient. Further research is essential to assess a large number of reliable predictors offering the opportunity to anticipate the individual risk for AF/AT recurrence following catheter ablation. Previous data suggests that LA-remodeling plays an important role for AF/Atrial tachycardia (AT)-recurrence after PVI. However, LA remodeling is an electrical and anatomical process and therefore difficult to measure directly [5,6]. In this context there is already evidence that the type of AF, LA-dimensions, LA-anatomy and LA-volume may be related to left atrial remodeling and might therefore have the potential to act as significant predictors for AF recurrence after PVI [7,8]. In addition, a recent study discussed that elevated left atrial pressure (LAP) depicts a possible trigger for AF by causing ectopic beats emanating from the pulmonary veins (PVs) [9].

It is still unclear whether elevated LAP has a significant effect on freedom from AF/AT recurrence after PVI and whether this physiological parameter relates directly to anatomical and structural changes of the LA. Our aim was therefore to prospectively analyze if the quantification of LAP is associated with the outcome following PVI.

## Methods

### Patient selection

120 consecutive patients with drug-refractory, symptomatic paroxysmal or persistent AF were included in this study. All patients underwent PVI between November 2009 and April 2012 at our clinic. All interventions were performed with at least one well-experienced electrophysiologist and usually one or two cardiologists in training. Every patient underwent circumferential isolation using radiofrequency (RF) lesions. All clinical and procedural data were prospectively recorded. Written informed consent was obtained from each patient prior to the ablation procedure and the study was approved by the institutional review board. According to the HRS consensus paper from 2007, paroxysmal AF was defined as self-terminating episodes lasting less than 7 days. Persistent AF was defined as AF lasting more than 7 days, and/or requiring electrical or pharmacological cardioversion [10]. Exclusion criteria were hyperthyroidism, LA thrombus, decompensated heart failure, stroke, myocardial infarction or gastrointestinal bleeding within 4 weeks prior to the intervention. Primary endpoint of this study was defined as long-term procedural success, defined as long-term freedom from any AT/AF episodes irrespective of symptoms after the index procedure during 12 months of follow-up. Secondary endpoints were procedure-related complications defined as death, atrio-esophageal fistulae, pulmonary vein stenosis requiring interventions, pericardial tamponade requiring intervention, phrenic nerve paralysis.

### Echocardiography

Transthoracic echocardiography (TTE) and transesophageal echocardiography (TEE) were routinely performed prior PVI. The TTE images were obtained from parasternal long- and short-axis views, apical four-chamber, two-chamber, and long-axis views. TEE was needed for exclusion of thrombus formation in the atria. All echocardiographies were performed according to the guidelines of the American Society of Echocardiography. [11]

### Multidetector computed tomography

All patients underwent ECG-gated 64-slice MDCT (VCT LightSpeed GE Healthcare, Milwaukee, WI, USA) within 24-48 h prior to PVA using our local LA protocol. [12] Briefly, heart rate was kept below 70 bpm using beta-adrenergic blocking agents if necessary. A spiral scan using retrospective gating was performed during a single breath-hold to examine the heart from the supraaortic region to the upper abdomen. In all cases, 80 mL of intravenous contrast agent (Imeron 350, Bracco Imaging, Konstanz, Germany) was injected. Semiautomatic bolus detection in the ascending aorta was used for optimal contrast timing. Imaging parameters included gantry rotation time of 350 ms, detector collimation of 64 x 0.625 mm, and a tube voltage of 120 kV. Semi-automatic dose reduction schemes were used. The radiation exposures range between 6.24 and 10.31 mSv. An ECG-gated half-scan algorithm was applied to reconstruct the data into axial images with a slice thickness of 0.625 mm. Ten phases within the cardiac cycle were reconstructed at 10% RR interval and the end-systolic phase of the LA selected as either 70 or 80% of RR length. MDCT images were analysed offline by experienced independent readers (at least one radiologist and one cardiologist) on a standard workstation with a dedicated cardiac imaging software package (VolumeShare 2, GE Healthcare). For each patient, anatomy of the LA, the PVs, and LAV were identified. As previously described, the two-dimensional LA area was manually traced on each MDCT slice from the LA roof to the level of the mitral annulus. The PVs were cut at the PV ostia and the LAA was excluded at its base. The LA volume was automatically calculated and reported. For the ablation procedure, 3D MDCT images of the LA and the PV were reconstructed on a separate workstation and integrated with electroanatomical mapping (CARTO Merge, Biosense Webster, Diamond Bar, CA, USA).

### Electrophysiological study and measurement of left atrial pressure

All ablation procedures were performed using a remote magnetic navigation system (RMN), the Niobe II magnetic navigation system (Stereotaxis) and a joystick-controlled motor drive (Cardiodrive, Stereotaxis). The RMN system has been described previously13. Access was achieved from the femoral veins in any cases. A 6F steerable decapolar catheter (Bard Dynamic Tip, Bard Inc., Lowell, MA, USA) was positioned in the coronary sinus. It was used as reference catheter during mapping and ablation process. After fluoroscopically guided transseptal puncture (TSP) a SL 1 sheath (St. Jude Medical, Inc., St. Paul, MN, USA) was inserted into the LA. At this point the LAP was measured invasively using the monitored pressure curve. The maximum LA pressure (mLAP) was defined as the maximum height of the v wave, and the minimum LA pressure (miLAP) was defined as the minimum of the x trough in case of SR during measurement or the lowest value of the pressure curve in the case of AF during measurement. After this measurement, a 3.5 mm open-irrigated, magnetic mapping and ablation catheter (Navistar Thermocool RMT, Biosense Webster, Diamond Bar, USA) was advanced through the sheath into the LA. Mapping was performed using a 3D-mapping system (CARTO Merge, Biosense Webster, Diamond Bar, CA, USA). The radiofrequency (RF) generator (Stockert, Biosense Webster) was set to temperature controlled RF delivery with a target temperature of 45ºC and a nominal power limit of 40 W (flow 30 ml/min). At the posterior LA wall the output was limited to 30 W (flow 17 ml/ min). RF current was applied for 30-60 s until local electrogram amplitude was reduced by 80%. Endpoint of the ablation procedure was the electrical isolation of all PVs defined as bidirectional conduction block. Conduction block was confirmed by careful and repeated mapping for residual potentials around the entire circumference of the PV ostia, and pacing from multiple sites within the circumferential line. For this purpose, the entire ablation line was mapped after the first anatomical circumferential attempt to detect residual potentials representing local conduction. If necessary, additional ablation lesions were added. Afterwards, the complete ablation line was re-mapped again during pacing from the distal pair of electrodes of the RMN catheter with an output of 10 V/2 ms (Biotronik UHS 3000 Control Unit, Biotronik GmbH) and the presence or absence of LA capture was evaluated using the atrial signal of the coronary sinus catheter. If no capture was detected, the line was considered as complete at this specific location. In case of LA capture a gap was suspected and ablation was continued [14,15].

An echocardiogram was performed within 24h after index procedure to detect pericardial effusion.

### Follow-up

Daily 12-lead surface ECGs and telemetry until discharge were used to confirm sinus rhythm. Additional antiarrhythmic drug therapy was prescribed for the first 3 to 6 months if necessary and oral anticoagulation was started the day after PVI with a target INR of 2.0-3.0. Bridging with unfractionated or low molecular weight heparin was initiated 6-12 h after the ablation procedure. After hospital discharge, all patients were scheduled in our outpatient clinic 3, 6, 9 and 12 months following PVI. Upon every visit, patients were asked for symptoms of AF/AT recurrence, documented arrhythmia recurrences, and current medication. Moreover, ambulatory Holter monitoring was performed at each visit for 96h to detect symptomatic or asymptomatic AF/AT recurrences. All patients were advised to present themselves immediately in the case of symptoms suggestive of AF/AT recurrence in order to obtain necessary treatment and ECG documentation. An AF/AT episode lasting longer than 30 s outside a blanking period of 3 months after the index procedure was considered as recurrent AF/AT.

### Statistical analysis

Statistical analysis was performed using SPSS for Windows (Version 20.0, SPSS Inc., Chicago, IL, USA). Continuous variables are expressed as mean ± standard deviation. Normally distributed data were compared using the independent Student's T-test. A P-value < 0.05 was considered as statistically significant. A Kaplan-Meier (KM) analysis of the median value of a continuous parameter was used as the standard cutoff value. Freedom from AF/AT recurrence for the dichotomized patient groups was compared using logistic regression. Multivariate logistic or Cox regression analysis was performed to assess those factors that achieved significance in univariate analysis. Receiver operating characteristic (ROC) analysis was performed to assess the predictive value of parameters for ablation success. The area under the ROC-curve (AUC) as well as the asymptotic significance were calculated. A linear regression model was used to determine linear correlations. Results are given with Pearson's "R". The Spearman Rho coefficient was calculated to spot non-linear correlations.

## Results

### Patient details

A total number of 120 consecutive patients, aged 63 ± 8 years, including 168 ablation procedures were analyzed. The clinical baseline characteristics of the entire study population are presented in Table 1. In all patients at least one antiarrhythmic drug failed prior catheter ablation. Those were amiodarone (43%), dronedarone (33%), and flecainide (58%). Most of the patients had paroxysmal AF (58 %). All patients underwent TTE, TEE and MDCT prior PVI without complications.

### Left atrial pressure

**Figure 1:**
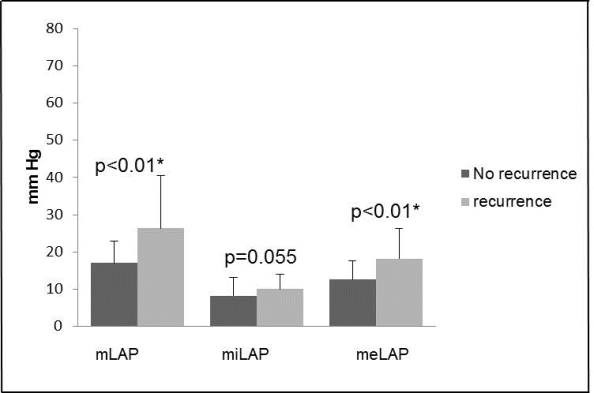
Comparison of different left atrial pressure (LAP) measurements between patients with and without AF/AT recurrence. The level of LAP (mmHG) is displayed on the Y-axis. The figure demonstrates that mLAP and meLAP are significant elevated in patients with AF/AT recurrence (p<0.01). The miLAP shows a strong tendency to the level of significance too (p=0.05).

Maximum LAP (mLAP) and mean LAP (meLAP) were significantly higher in patients with AF/AT recurrence (mLAP p<0.01; meLap p<0.01). Furthermore the minimum LAP (miLAP) showed a strong tendency to the level of significance (miLap p=0.05; Figure 1). We observed a significant difference (p=0.047) regarding the amount of mLAP comparing patients with paroxysmal (PAF) and persistent AF (PERS).

### Procedural success and AF recurrence

**Figure 2:**
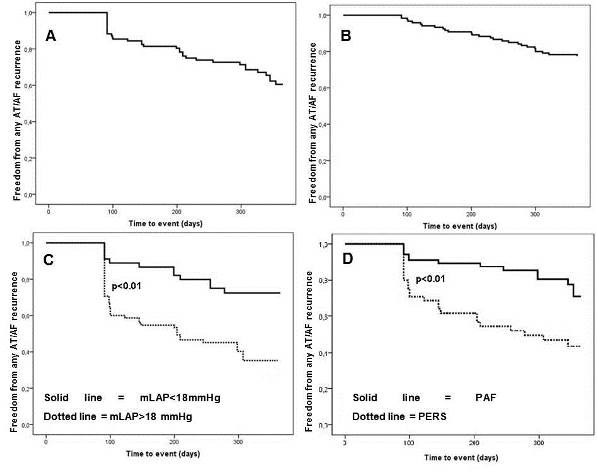
A AF/AT recurrence-free survival after one ablation procedure. B Overall survival after 1-year including addition ablation procedures. C Kaplan-Meier analysis of the mLAP >18 mmHg (dotted line) vs the mLAP <18 mmHg (solid line). The outcome is significantly lowered in the group with a mLAP >18 mmHg (p<0.01). D Kaplan Meier analysis of patients with persistent atrial fibrillation (AF) (dotted line) vs those with paroxysmal AF (solid line). The recurrence free intervall is significantly lowered in patients with persistent AF (p<0.01).

The mean follow-up period was 303±94 days. At the end of this period 60% of the patients maintained sinus rhythm after the initial ablation procedure and 78% after additional PVI (including 1.4±0.5 ablation procedures, Figure 2).

In univariate analysis (using Cox-Regression) type of AF, LA-Volume, congestive heart failure (CHF), mLAP and meLAP were significantly correlated with AF/AT recurrence after PVI (p=0.03; p=0.001; p=0.01). Multivariate analysis showed that type of AF, LA-volume and mLAP are independent predictors for recurrence (p=0.017; p=0.019; p=0.001). Performing a ROC-curve with maximum and meLAP, both curves reached the level significance of p<0.01. The AUC for the mLAP was higher than the one from meLAP (AUC=0.75 vs AUC=0.73). A mLAP greater than 18 mmHg predicts recurrence with a sensitivity of 77 % and specificity of 60 %. Plotting the type of AF in a KM-Curve it reveals a significant difference between paroxysmal AF and persistent AF (Figure 2D; log-rank p=0.02). Our data shows that a mLAP>18 mmHg is associated with a Hazard ratio (HR) of 3.8 (Fig 2C; log-rank: p<0.01). Moreover, the presence of persistent AF is associated with a HR of 2.0 for AF/AT recurrence in multivariate analysis. In addition, LAV more than 100 ml goes within a HR of 2.1 for recurrence (Table 2).

There were no major complications requiring intervention. Pericardial effusion (n=2) and haematoma at the puncture site (n=1) were documented as minor complications, none of these required intervention.

### Correlation between LAP and LA dimension

**Figure 3:**
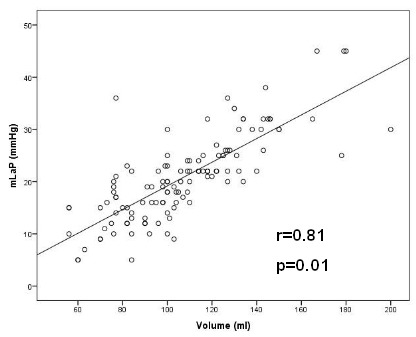
Linear regression model between the LAV (ml) on X-axis and the mLAP (mmHg) on Y-axis. Pearsons r is 0.81 (p=0.01). Whereas the other two pressure parameters did not show sufficient linear correlation to the LAV. There was also a linear correlation between persistent AF and mLAP (p=0.034; r=0.19; Figure 4).

Spearmans rank correlation was performed to examine if there is a direct correlation between LAP and other clinical characteristics. Linear regression analysis showed a significant correlation between the LAV and the mLAP (r=0.81; p=0.01*) which is demonstrated in Figure 3.

### Subgroup analysis

According to the multivariate regression model patients were categorized into 4 groups. Patients were scheduled depending on the following characteristics: PERS, LAV > 100 ml and mLAP >18 mmHg. Patients with none of these three characteristics were summarized in group 1 (n=24). Group 2 (n=28) consistent of patients with 1 out of the 3 factors. If 2 out of 3 factors were positive, patients were categorized in group 3 (n=38). In case that all 3 out of 3 factors were present, patients were categorized in group 4 (n=30). The patient characteristics of each patient group are presented in Table 3.

**Figure 5:**
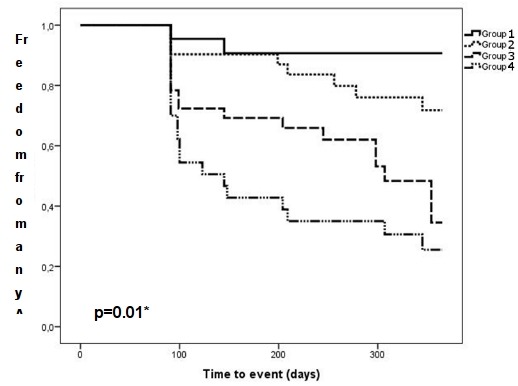
Kaplan-Meier analysis of the 4 subgroups. The figure demonstrates that the more risk factors (persistent AF (PERS), left atrial volume (LAV)>100ml, maximum left atrial pressure (mLAP)>18 mmHg) are positive, the higher is the possibilty for AF/AT recurrence (p=0.01).

Figure 5 shows the KM analysis for the 4 patient groups. After the mean follow up of 303±94 days, 92% of the patients in group 1 were free of any recurrence after a single procedure. After the same period, there were 68% of the patients recurrence-free in group 2. In group 3 38% and in group 4, where all characteristics were present, 26% were free of recurrence.

## Discussion

### Main findings

The main finding of this prospective study is that mLAP and meLAP are good predictors for AF/AT recurrence after catheter ablation for AF (p<0.01). In multivariate analysis, an mLAP >18 mmHg was significantly associated with an elevated risk for AF/AT recurrence (HR 3.8). The mLAP shows a good linear correlation to LAV (p=0.01). Furthermore, mLAP is associated with a higher probability for the presence of persistent AF (p=0.03). In addition, mLAP>18 mmHg seems to be in close relationship to the presence of congestive heart failure, too (p=0.05).

### The role of risk factors and LA remodeling to predict AF/AT recurrence

It is still of emerging interest, to find clinical predictors for AF/AT recurrence after PVI. Over the years, a lot of different predictors have already been described. Recent data suggests that LA dimensions relate to the success rate of the ablation procedure. Several studies have demonstrated that a LAV >100 ml significantly increases the risk for AF/AT recurrence after PVI9,16-18. Despite the LAV, the type of AF is a strong predictor for a successful ablation procedure, too19. In this context, the presence of persistent AF is associated with a poorer prognosis in comparison with paroxysmal AF20. Our results are in line with previous data, demonstrating that the LAV was a significant predictor in multivariate analysis (p=0.019, Table 2). Furthermore, the presence of persistent AF was associated with a significantly increased risk for recurrence (p=0.017, Table 2). Our data adds valuable information regarding the role of LAP in the context of AF/AT recurrence. We indicate that the mLAP was significantly associated with an increased risk for recurrence after PVI (p<0.01; HR=3.8; Table 2). Comparing the AUC, the mLAP showed a better correlation with AF/AT recurrence than the meLAP (AUC mLAP=0.75 vs AUC meLAP=0.73). These findings are in line with results, which have been published recently [21,22]. The studies also evaluated the role of LAP in context of AF/AT recurrence after PVI. The results of those studies corroborate the emerging role of LAP on freedom from AF/AT recurrence. In contrast to the other studies, PVI was performed using a remote magnetic navigation system (RMN) in our study.

Based on these findings we suggest that all the three factors play an important role in the process of LA remodeling in patients with AF. Besides the anatomical remodeling, which can be visualized, there is evidence for additional electrical and physiological changes of the LA in patients with AF. In previous studies it could be shown that elevated LAP leads to changes in the electrical velocity of the myocardium with the potential to generate delay [23]. Those conduction delays may trigger micro-re-entry. In addition, it has been demonstrated that elevated LAP increases the rate of waves emanating from the PVs [8] and an experimental study showed that an increase in LAP results in the development of AF [24]. This is in line with our findings and therefore we suggest to deem the elevated LAP as an important predictor for freedom from AF/AT recurrence after PVI.

Furthermore, there is evidence, that ACE inhibitors may play a role in preventing AF [25,26]. As shown before Angiotensin II leads to an increase in LAP [27,28]. The protective effect of ACE inhibitors on the development of AF might be in the prevention of elevated LAP and therefore decelerating left atrial remodeling. This finding supports the importance of LAP in atrial remodeling.

### Correlation between LAP and left atrial dimensions

Previous data suggests that an enlargement of the LA relates to the level of LA remodeling. In this context, the elevation of the LAV has been shown as suitable predictor for AF/AT recurrence in several studies [16-18]. This study demonstrated a good linear correlation between the mLAP and meLAP to the LAV (rs=0.80; rs=0.76). Especially the mLAP showed a clear linear correlation with LAV (r=0.75, Figure 3). These findings could be explained by several reasons: First, the loss of the regular atria contraction during AF might lead to a severe volume overload in the LA and this might increase the LAP. The correlation between LAP and LAV has been reported earlier, our results confirm these findings [29,30].

Second, the presence of CHF showed a significant correlation with the miLAP and the meLAP. Independently of mitral valve diseases the presence of CHF and diastolic dysfunction is said to be associated with increased LAP [31]. Furthermore atrial fibrosis may lead to a decreased ability of the atrium to relax resulting in a dysfunction similar to the diastolic dysfunction of the ventricle [32]. This could be an explanation for the correlation of the miLAP as an expression of the loss of atrial distensibility.

Third, the presence of persistent AF correlated significantly with mLAP (p=0.017, rs=0.190). This finding alone is not surprising, as the other correlated factors can act as a precondition for the development of persistent AF [33]. The correlation of LAP with the LA-volume is highly significant. In addition, experimental models showed as well that even the induction of AF goes within elevation of LAP, independently of structural heart diseases [34].

### Risk stratification for AF recurrence

Even for the experienced electrophysiologist, it remains challenging to calculate an individual risk for AF/AT recurrence after PVI. We want to present an approach to categorize patients according to their clinical risk factors and calculate their individual risk based on cut off values. Our study demonstrates that a lot of risk factors correlate with each other. By combining these factors it was possible to subdivide the patient group in different risk categories. We used an mLAP of >18 mm HG and a LAV >100 ml as cut-off values based on the ROC. As shown in Figure XY/Table XY, the probability for freedom from AF/AT recurrence decreases, the more factors have reached the cut-off value. Patients categorized to group 4 have a relatively low probability for mid-term freedom from AF/AT recurrence of only 24%.

For accurate use of the scoring-system, an invasive measurement of LAP is required, which is usually performed at the time of performing PVI. In order to have an unambiguous scoring before the procedure it might be useful to identify imaging based parameters e.g. in CT or echocardiography, that correlate with the LAP.

Nevertheless, the demonstrated risk score based on clinical parameters could be an alternative to the recently publish risk stratification model by Marrouche et al . They suggested to predict the success rate of PVI based on pre-procedural fibrosis detected on MRI. In their study, atrial fibrosis was taken as expression of left atrial remodeling [35]. In contrast to this study, we used parameters, which are ascertained routinely prior PVI. In addition our statistical calculation is less time consuming and more cost-effective than using an MRI-based risk stratification alone. However, our data shows that our approach of individual risk stratification is feasible and lead to reproducible results. We would therefore suggest including this cut-off based model into clinical routine.

### Risk stratification for AF recurrence

## Conclusion

LAP is a parameter easy to access during PVI. The maximum LAP displays a significant predictor for recurrence after PVI. There is a good correlation between LAP and LA-volume and both factors may be useful to quantify LA remodeling.

## Figures and Tables

**Figure 4:**
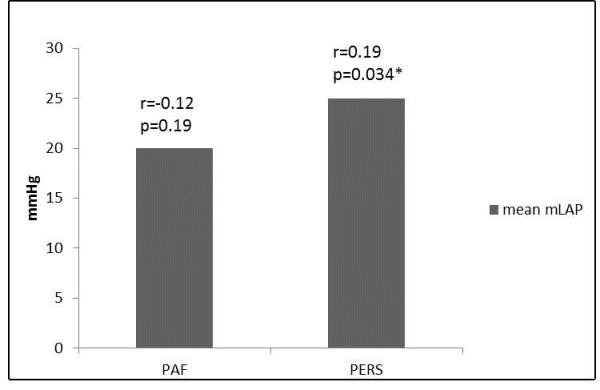
Bar graph displaying the correlation of maximum left atrial pressure (mLAP) to the type of AF. The mean mLAP is higher in patients with persistent atrial fibrillation (PERS; r=0.19; p=0.034) in contrast to patients with paroxysmal atrial fibrillation (PAF; r=-0.12; p=0.19).

**Table 1:**
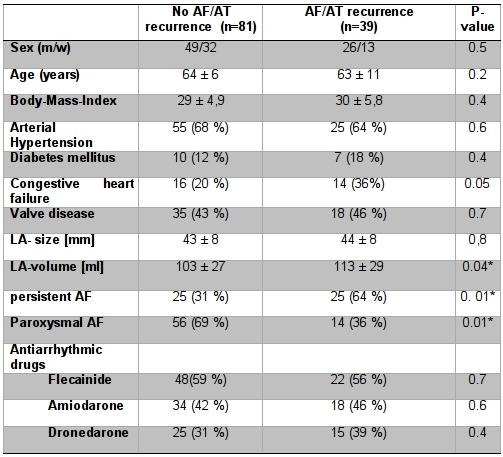
Clinical parameters of all patients

P-Values are given for all parameters. LA volume (LAV) and type of atrial fibrillation (AF) differ significantly between patients with and without AF/AT recurrence (*=significant).

**Table 2:**
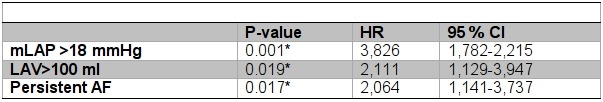
Summary of the significant predictors in multivariate regression analysis

(mLAP=maximal left atrial pressure; LAV=left atrial volume: AF=atrial fibrillation; CI=confidence interval; *=significant).

**Table 3:**
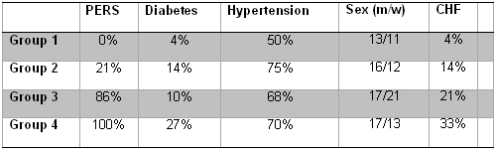
Patient details divided into the different subgroups

(PERS= persistent atrial fibrillation; congestive heart failure=CHF).
